# A Call for Emergency Action: Telepsychiatry for Trauma Treatment Among Syrian Refugees

**DOI:** 10.7759/cureus.1578

**Published:** 2017-08-18

**Authors:** Muhammad Aadil, Rosario M Cosme, Fernando E Forcen, Ahmad R Khan

**Affiliations:** 1 Department of Medicine, FMH College of Medicine and Dentistry; 2 Department of Child Psychiatry, Rush University Medical Center; 3 Department of Psychiatry, Rush University Medical Center; 4 Department of Psychiatry, University of North Dakota

**Keywords:** telepsychiatry, ptsd, post-trauma, syria, mental health

## Abstract

The war in Syria has entered the seventh year, with no sign of resolution. It is often referred as "the greatest human tragedy" since World War 2. Because of limited health facilities, we propose telepsychiatric interventions for the provision of mental healthcare services in Syria and in refugee camps to treat post-trauma patients.

## Editorial

The civil war in Syria, which started in 2011, resulted in an estimated half-a-million deaths with over four-million refugees and seven-million internally displaced persons. This war has lasted for seven years, and it is possibly the largest humanitarian crisis since World War II. The conflict has resulted in a massive displacement of Syrian citizens, both internally and externally; they need psychiatric help. The influx of Syrian refugees in Turkey, Lebanon, and Middle Eastern countries has resulted in a shortage of medical doctors in those countries as well. We want to highlight the importance of telemedicine in such areas, primarily to treat trauma-related disorders, which are prevalent among Syrian refugees and probably neglected because of limited healthcare facilities.

Refugees are at a higher risk of psychiatric comorbidities, including post-traumatic stress disorder (PTSD) [1]. A recent study conducted on Syrian refugees in Lebanon, which is the leading host country for Syrian refugees, showed that out of 452 respondents, the lifetime prevalence of PTSD was 35.4% with a point prevalence of 27.2% [1]. The factors behind this can be various, including experiencing tragedies and traumatic events, forced migrations, and relocating to new socio-cultural locations. Another study conducted in Turkey to study the prevalence of PTSD among Syrian refugees showed that the incidence is 35.5% [2]. The study concluded that PTSD is as high as 71% if the refugees had the following features: female gender, prior diagnosis of a psychiatric disorder, a family history of psychiatric disorders, and the experience of two or more traumas [2]. Though the prevalence of PTSD in different studies varies, which may be due to various factors, including different methodologies, sample size, and clinical and political factors, more epidemiological studies are required, especially in Syrian refugees.

Syria and most Arab countries providing refuge to Syrians have limited psychiatry health providers. Seven Arab countries (Iraq, Libya, Morocco, Somalia, Sudan, Syria, and Yemen) have less than 0.5 psychiatrists per 100,000 population [3]. This shows how understaffed and ill-equipped these regions could be in providing proper mental health care to patients who have PTSD and other psychiatric problems. Despite the efforts made by the international community to provide mental health to Syrian refugees, data is too limited to know the exact impact. According to a study funded by the World Health Organization (WHO) and International Medical Corps (IMC) in collaboration with the Jordanian Ministry of Health and Eastern Mediterranean Public Health Network (EMPHNET ), it was found that only 13.5% of the refugees in Jordan refugee camps have access to mental health treatment and psychosocial support.

In such circumstances, it might now be time to start using new technologies like telemedicine to provide mental health care to Syrian refugees, especially those living outside of refugee camps with no access to healthcare providers. Telemedicine consists of providing health care from a distance through technology, often using video conferencing. Telepsychiatry, a subset of telemedicine, can involve providing a range of services, including psychiatric evaluations, therapy (individual or group therapy), patient education/counseling, and medication management. It comes with benefits that include reduced cost, reduced barrier of stigma, improved access, continuity of care, and follow-up. The latest data shows that telepsychiatry has been found to be equally effective in treating PTSD compared to the usual treatment. A study conducted on the veterans of Iraq/Afghanistan who received cognitive processing therapy through video-conferencing for PTSD was found equally effective to the treatment delivered in person [4]. Hussam Jefee-Bahloul conducted a pilot study to look for the openness of Syrian refugees suffering from PTSD. Thirty-four percent of the whole sample reported a perceived need to see a psychiatrist, and among them, 45% were open to receive telepsychiatry care [5].

Keeping a view of the cultural barriers, limited access to mental health services, limited funding, political crisis, and a new surge in war crimes, it is important to make arrangements to provide mental health care for the treatment of trauma-related disorders in both refugee and war-struck zones. The establishment of programs to provide telepsychiatry care would need cooperation and funding from international organizations like WHO and the United Nations (UN) and can be started on an empirical basis, at least in Turkey and Lebanon, which are hosting a vast majority of the Syrian refugees. More studies are also required to understand the cultural and social barriers limiting the use of telepsychiatry care in these regions. Despite the lack of studies and understanding, telepsychiatry appears to be a very cost and clinically effective approach to care for persons suffering from PTSD in the war-struck zones of Syria.
